# High sensitive label-free optical sensor based on Goos–Hänchen effect by the single chirped laser pulse

**DOI:** 10.1038/s41598-020-74212-8

**Published:** 2020-10-14

**Authors:** Elnaz Rezaei Benam, Mostafa Sahrai, Jafar Poursamad Bonab

**Affiliations:** 1 Department of Laser and Optical Engineering, University of Bonab, Bonab, Iran; 2 Faculty of Physics, University of Tabriz, Tabriz, Iran

**Keywords:** Sensors and biosensors, Imaging and sensing

## Abstract

We consider a four-level molecular system with two ground-state vibrational levels and two excited-state vibrational levels inside a constant cavity configuration. We discuss the reflected and transmitted Goos–Hänchen (GH) shifts of a positive and negative single-chirped laser pulse. The impacts of the laser field detuning, intensity of applied laser field, and appropriately tuning the chirp rate on GH shifts are then analyzed. It is also found that this sensor is very sensitive to the refractive index of the intracavity medium, which can coherently be controlled by the medium parameters. The results show that such a sensor can be most effective for detecting biological molecules with low concentration than the large number density, where a bit variation in the concentration of sample will lead to a great variation on the GH shifts.

## Introduction

It is well known that the biological and chemical samples can be recognized by optical biosensors. In fact, a biosensor senses a sample and produces a physical or chemical response. Thus, they are commonly utilized to detect biomolecules that either represents a disease or a drug target. Biosensors can also be used for environmental monitoring^1^, disease diagnosis^2^, food safety^3^, drug discovery^4^. Among various detection possibilities, using the surface modes sensing is an accurate approach. Usually, an optical sensor accompanies by a total reflection, which produces the surface mode^5^. Also, the reflected light is extremely sensitive to the layer thickness, and the optical properties of the surface, such as the refractive index. So, the shift of the reflected light gives a quantitative measurement upon the properties of the surface layer. Based on recent observations, phase-related measurements are more sensitive. Phase-related measurement completely depends on the molecule concentration in the interface of two media. Thus, this has been employed for label-free detection of biological species. Among the investigation of various proposals, phase-detection method is more sensitive than the method based on intensity detection^6^.

Sreekanth et al.^7^ used a four-layered metal-dielectric system with a highly absorbing thin layer of germanium. They experimentally showed that this optical system presents a large phase change at the point of darkness at the Brewster angle. In another study, Sreekanth et al.^8^ presented a novel technique for fabricating silver–stibnite nonporous plasmonic films and investigated the existence of propagating surface plasmon polaritons in nanoporous films. They also employed the Goos–Hänchen shift interrogation scheme and showed the extreme bulk refractive index sensitivity of the films. They also, in another study, reported a reconfigurable plasmonic biosensor platform based on active Sb2S3–TiN HMMs for small molecule detection at low concentrations^9^. Yan et al.^10^ proposed a lithography-free sensing platform based on the metal-dielectric cavity. They found that this sensor has excellent sensing performance in both TE and TM light, and suitable for integrated microfluidic channels.

When an optical beam radiates upon an optical layer, the reflected beam shall laterally be shifted in the plane of incidence. This is called the Goos–Hänchen (GH) effect^11,12^. In fact, the Goos–Hanchen shift is the small displacement of a light beam when it is totally internally reflected at the interface of two media. In this optical phenomenon, the incident light penetrates first into the lower refractive index medium before being totally reflected and back into the high index medium. This is also known as an ‘evanescent wave’. The interaction of this evanescent wave with refractive index changes can be utilized for sensing purposes. The reflected GH shift at the interface provides the appropriate phase information that can be observed in various configurations.

The GH effect has attracted a lot of attention for discovering the profound physical meaning behind it, and also potential applications. Practical applications of the GH shifts include the design of an optical heterodyne sensor^13^ or an optical waveguide switch^14^, bio-sensing technologies^15^, and detection of chemical vapors^16^. The GH shift is very sensitive to the property and geometrical characteristics of the constituents of the optical system. Thus, it can be used for measuring the medium parameters such as refractive index, beam apertures, and incidence angles, temperature, displacement, or film thicknesses. Measurement based on GH shifts for such quantities is simple, secure with high sensitivity and large dynamic range. Therefore, sensors based on GH shifts could be used for industrial applications. Chen et al.^17^ reported an optical sensor with a metal–dielectric interface for temperature sensing based on the GH effect. They found negative GH shifts as a function of the temperature for p-polarized incident light at grazing incidence upon the metal. Nie et al.^16^ presented a symmetrical metal-cladding waveguide with a polymer layer in guiding layer, for sensitive detection of chemical vapor by using the enhanced GH shift. In their proposal, the relative GH shift linearly responds to the chemical vapor concentration, and the phase-matching condition is changed by the refractive index variation in the polymer layer. Recently, Yin et al.^18^ introduced a terahertz biosensor based on the GH effect in grapheme. According to their report, this sensor with a sensitivity of $2.5\times {10}^{4} \,\upmu{\mathrm{m}}/\mathrm{RIU}$ can be used for detecting the small biomolecules.

Here, a label-free sensor based on GH shifts of the reflected and transmitted laser field is proposed. We introduce three layers I, II, and III, where layers I and III are the walls of the cavity with the same nonmagnetic dielectric substance. Layer II includes a four-level molecular system with frequency-dependent susceptibility. Basically, a small portion of the incident laser field penetrates through the reflecting medium. The transmitted wave can interact with the medium, including a four-level molecular system. It is observed that the optical susceptibility of the medium can be changed by the system parameters and the intensity of the laser field. This variation modifies the resonant condition of the cavity, and therefore the manipulation of the field on the lateral shifts could be observed. Here, we use a single-chirped laser pulse that couples ground vibrational states to excited vibrational states. Then the absorption, reflection, and transmission behaviors of the incident light are analyzed. To show the advantage of the proposed model with a single-chirped laser pulse, Zhang et al.^19^ discussed the population transfer and the creation of the coherent quantum states in a Λ-type excited-doublet four-level system. They employed the time-dependent Hamiltonian for the system in the rotating-wave approximation and solved the time-dependent Schrödinger equation to discuss the time evolution of the system. Their results show that the population in the primary state can be transferred to any of the states based on the chirped adiabatic passage (CHIRAP). This is a simple and supple way compared to other methods, which utilizes two or more laser pulse for the creation of the arbitrary superposition of quantum states in multilevel systems.

In another study, Wan et al.^20^ demonstrated a polarization-modulated GH shift sensing system for common-mode noise and drift suppression that is experimentally confirmed for a Bloch surface wave sensor.

Then, we investigate the behavior of the GH shift and reflectivity versus chirped laser field detuning for positive and negative chirp spectrum. Since the amount of GH shift is typically on the order of wavelength, a bit variance of the structure parameters can lead to a demonstrative variation in the GH shift. So, this tunable property should be an engaging method to plan modern sensing devices.

The major advantage of using GH shifts is the ability to couple light into the surrounding medium, which suggests a large interaction surface. Also, the amount of beam shift could be measured directly in experiments using a simple setup, compared to complicated interferometry setups for phase measurement^21^. Based on recent observations, there is a linear relationship between the amount of GH shifts and the concentration of samples^22^. Note that Sun et al.^22^ utilized the symmetrical metal-cladding waveguide structure for detecting E. Coli O157:H7. Using a metal cladding waveguide, the concentration of the bacterial pathogens on the enhanced Goos–Hanchen shift is experimentally proposed.

So, we alter the concentration of the sample and study reflectivity and the GH shift of the reflected single-chirped laser pulse. Furthermore, we can control the coherent superposition of the states by proper detuning of the laser field and tuning the chirp rate. The penetrate depth of the laser field versus concentration of the sample has also been studied. Note that the presented proposal is different.

In this proposal, we introduce a label-free optical sensor based on the GH effect with a single-chirped laser pulse, which is not already reported in a four-level molecular system with a positive and negative chirped laser pulse according to our acknowledge. For this purpose, the response of the four-level molecular system to the single-chirped applied laser field is investigated. The chirp spectrum of the applied laser field along with the intensity of the field is then employed to control the susceptibility of the intracavity medium and consequently, the GH shifts of the incident laser field. Influence of the sample concentration on GH shifts, reflectivity, and penetrate depth is also studied. This report will provide a model to determine the characteristics of the molecules, and will introduce an effective mechanism for designing the optical sensing devices. Thus this will improve the sensitivity of the label-free sensors.

The rest of this paper is as follows; in the following section, we introduce the model and the related equations of motion. The detail of the calculations is also presented in this section. We analyze the results in "Results and discussion" section, and provide a brief conclusion in "Conclusions" section.

## Model and equations

**Figure 1:**
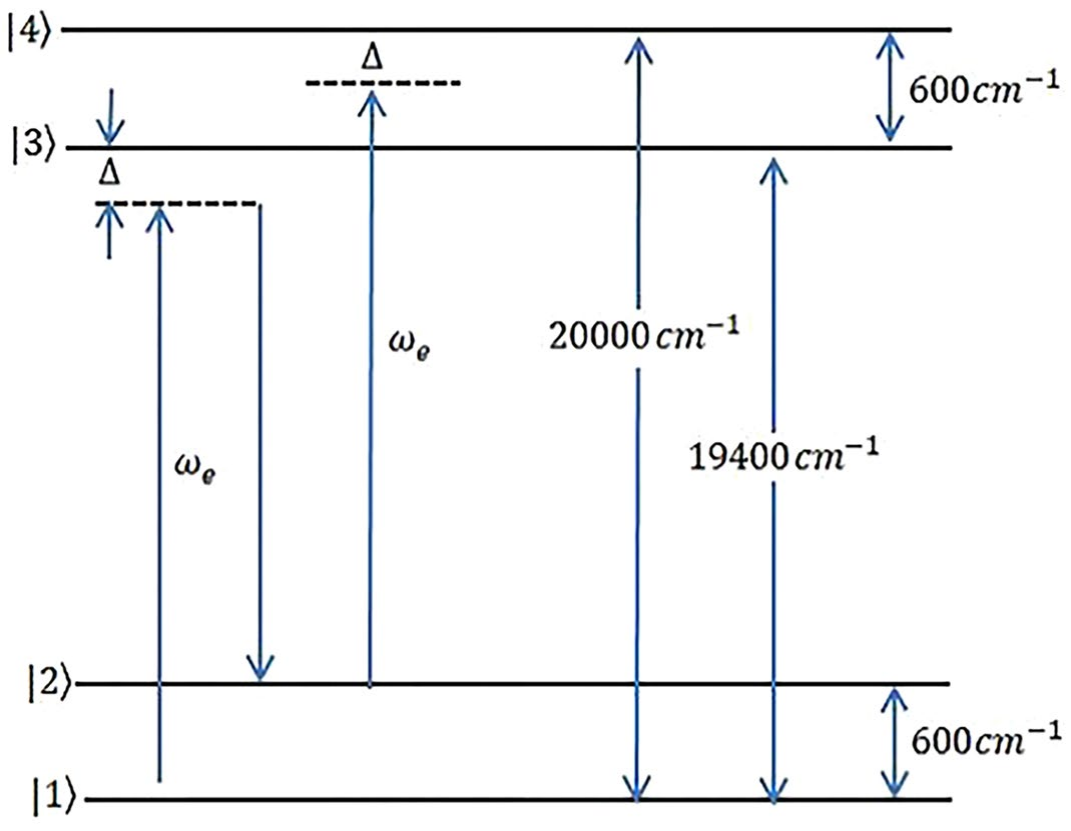
Four- level molecular system with two vibrational levels in ground state and two vibrational levels at excited state that interact with a single chirped laser field.

Propose a four-level molecular system with two ground-state vibrational levels and two excited-state vibrational levels interacting with a single-chirped laser pulse (Fig. 1). The linear-chirped electric field is defined as

1 $$E\left(t\right)=A\left(t\right){e}^{-i\omega t}+c.c.$$

where

2 $$A\left(t\right)={E}_{0}{e}^{{t}^{2}/{\tau }^{2}}{e}^{i\varphi (t)}.$$

Here, $$ \omega $$, $$ A\left(t\right)$$, are the central frequency and amplitude of electric-field,$$ {E}_{0}$$, and $$\tau $$ are the amplitude peak value at time $$t=0,$$ and transform-limited pulse duration, respectively. $$\varphi \left(t\right)\left(=\beta {t}^{2}\right)$$ represents the slowly varying phase with linear chirp rate $$\beta $$ that is chosen constant for transform-limited pulses. The density matrix equations of motion in a proposed four-level molecule in rotating wave approximation are obtained as^23^

3 $$ \begin{aligned} \dot{\rho }_{11} & = - \dot{\rho }_{22} - \dot{\rho }_{33} - \dot{\rho }_{44} . \\ \dot{\rho }_{22} & = - \gamma_{22} \rho_{22} + \gamma_{32} \rho_{33} + \frac{i}{2\hbar } \left\{ {\mu_{23} \left( {\rho_{23} E^{*} - \rho_{32} E} \right) + \mu_{24} \left( {\rho_{42} E^{*} - \rho_{24} E} \right)} \right\}. \\ \dot{\rho }_{33} & = - \gamma_{33} \rho_{33} + \gamma_{43} \rho_{44} + \frac{i}{2\hbar } \left\{ {\mu_{13} \left( {\rho_{13} E - \rho_{31} E^{*} } \right) + \mu_{23} \left( {\rho_{23} E - \rho_{32} E^{*} } \right)} \right\}. \\ \dot{\rho }_{44} & = - \gamma_{44} \rho_{44} + \frac{i}{2\hbar } \left\{ {\mu_{14} \left( {\rho_{14} E - \rho_{41} E^{*} } \right) + \mu_{24} \left( {\rho_{24} E - \rho_{42} E^{*} } \right)} \right\}. \\ \dot{\rho }_{12} & = - {\overline{\Gamma }}_{12} \rho_{12} + i\omega_{12} \rho_{12} + \frac{i}{2\hbar } \left\{ {\mu_{13} \rho_{32} E^{*} - \mu_{23} \rho_{13} E + \mu_{14} \rho_{42} E^{*} - \mu_{24} \rho_{14} E} \right\}. \\ \dot{\rho }_{13} & = - {\overline{\Gamma }}_{13} \rho_{13} + \left( {i\omega_{31} - \omega } \right)\rho_{13} + \frac{i}{2\hbar } E^{*} \left\{ {\mu_{13} (\rho_{33} - \rho_{11} ) + \mu_{14} \rho_{43} - \mu_{23} \rho_{12} } \right\}. \\ \dot{\rho }_{14} & = - {\overline{\Gamma }}_{14} \rho_{14} + \left( {i\omega_{41} - \omega } \right)\rho_{14} + \frac{i}{2\hbar } E^{*} \left\{ {\mu_{14} (\rho_{44} - \rho_{11} ) + \mu_{13} \rho_{34} - \mu_{24} \rho_{12} } \right\}. \\ \dot{\rho }_{23} & = - {\overline{\Gamma }}_{23} \rho_{23} + \left( {i\omega_{32} - \omega } \right)\rho_{23} + \frac{i}{2\hbar } E^{*} \left\{ {\mu_{23} (\rho_{33} - \rho_{22} ) + \mu_{24} \rho_{43} - \mu_{13} \rho_{21} } \right\}. \\ \dot{\rho }_{24} & = - {\overline{\Gamma }}_{24} \rho_{24} + \left( {i\omega_{42} - \omega } \right)\rho_{24} + \frac{i}{2\hbar } E^{*} \left\{ {\mu_{24} (\rho_{44} - \rho_{22} ) + \mu_{23} \rho_{34} - \mu_{14} \rho_{21} } \right\}. \\ \dot{\rho }_{34} & = - {\overline{\Gamma }}_{34} \rho_{34} + i\omega_{43} \rho_{34} + \frac{i}{2\hbar } \left\{ {\mu_{13} \rho_{14} E - \mu_{14} \rho_{31} E^{*} + \mu_{23} \rho_{24} E - \mu_{24} \rho_{32} E^{*} } \right\}. \\ \end{aligned} $$

where ${\mu }_{ij}$ are the dipole coupling coefficients of the electronic transitions, and ${\omega }_{ij}=\left|{E}_{j}-{E}_{i}\right|/\hslash$ are the angular frequencies of the transitions from level $i$ to level $j$.

The decay rates of the transitions $i-j$ are introduced by ${\gamma }_{ij}$, and the population relaxation rates of the levels satisfy ${\gamma }_{22}={\gamma }_{21}$, ${\gamma }_{33}={\gamma }_{31}+{\gamma }_{32}$, and ${\gamma }_{44}={\gamma }_{41}+{\gamma }_{42}+{\gamma }_{43}.$ The decay rates of the coherences are presented by ${\stackrel{-}{\Gamma }}_{12}={\Gamma }_{12}+{\gamma }_{22}/2$, ${\stackrel{-}{\Gamma }}_{13}={\Gamma }_{13}+{\gamma }_{33}/2$, ${\stackrel{-}{\Gamma }}_{14}={\Gamma }_{14}+{\gamma }_{44}/2$, ${\stackrel{-}{\Gamma }}_{23}={\Gamma }_{23}+\left({\gamma }_{22}+{\gamma }_{33}\right)/2$, ${\stackrel{-}{\Gamma }}_{24}={\Gamma }_{24}+\left({\gamma }_{22}+{\gamma }_{44}\right)/2$, ${\stackrel{-}{\Gamma }}_{34}={\Gamma }_{34}+\left({\gamma }_{33}+{\gamma }_{44}\right)/2$ which contain the purely dephasing rates ${\Gamma }_{ij}$ and relaxation of the coherences. The matrix elements ${\rho }_{13}, {\rho }_{14}, {\rho }_{23}$ and ${\rho }_{24}$ show the electronic coherences, while ${\rho }_{12}$ and ${\rho }_{34}$ are the vibrational coherences induced between the ground and excited states, respectively.

We use Laplace transformation to convert equations from time-dependent to frequency space. As a practical example, we choose an oxazine system with two ground-state vibrational levels and two excited-state vibrational levels. These systems possess important biological activities and are used in photochemistry and photobiology. We introduce the required parameters in equations as bellow^24^. Transitional dipole coupling coefficients and peak amplitude of field are set ${\mu }_{ij}=4.22\times {10}^{-29} \,\mathrm{cm}$,${E}_{0}=8.7\times {10}^{7} \,\mathrm{V}/\mathrm{m}$, respectively. The transition frequencies are defined as ${\omega }_{ij}=\left|{\varepsilon }_{j}-{\varepsilon }_{i}\right| /\mathrm{\hslash }.$ For the transitions $|1\rangle -|3\rangle$, $|1\rangle -|4\rangle  ,$ and $|2\rangle -|3\rangle$, wave numbers introduced as ${k}_{13}=2\pi \times 19400 \,{\mathrm{cm}}^{-1}$,${k}_{14}=2\pi \times \mathrm{20,000}\,{ \mathrm{cm}}^{-1}, { k}_{23}=2\pi \times \mathrm{18,800 }\,{\mathrm{cm}}^{-1}$, where ${k}_{ij}={\omega }_{ij}/c$.

**Figure 2:**
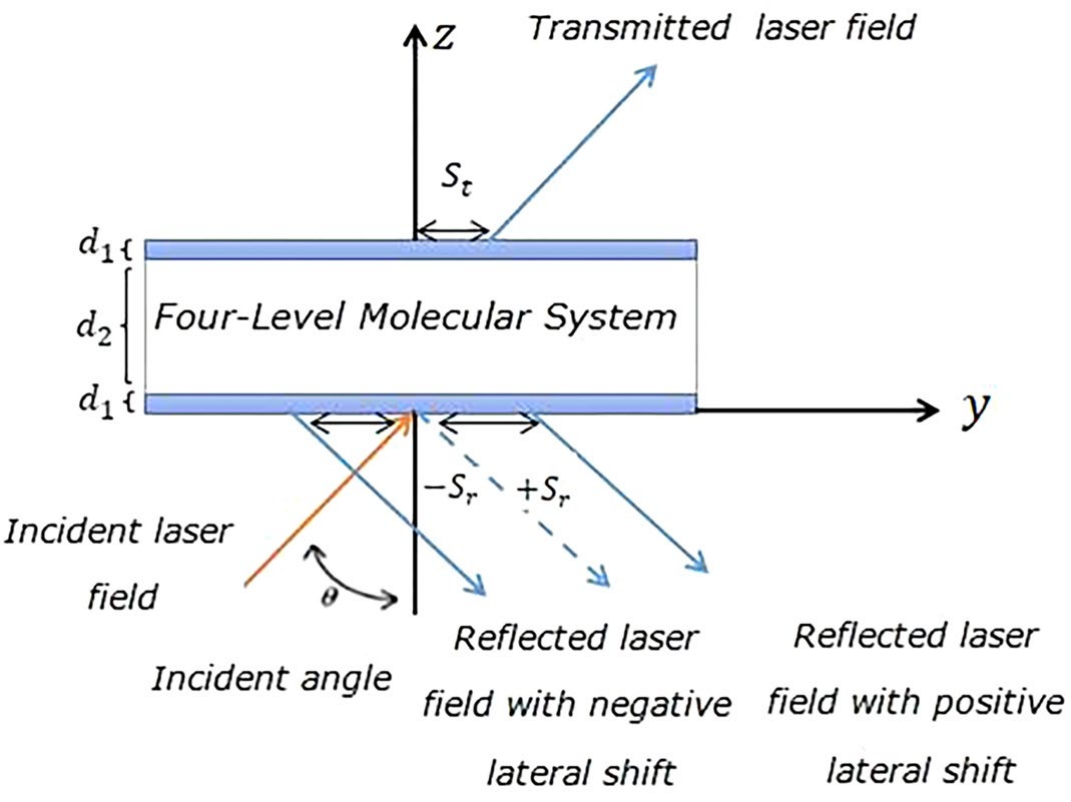
Schematic of a four- level molecular medium in a cavity with three layers (${d}_{1}, { d}_{2},{ d}_{1}$).The angle $\theta$ is the incident angle of laser field upon the cavity well along the $z$ axis, but ${S}_{r}$ and ${S}_{t},$ denote the reflected and transmitted GH shifts, respectively.

The wavenumber for the vibrational state is ${k}_{12}={k}_{34}=2\pi \times 600\,{\mathrm{ cm}}^{-1}$. The system is initially populated in the first vibrational level with ${\rho }_{11}=1$ and ${\rho }_{22}={\rho }_{33}={\rho }_{44}=0$. The population relaxation time for all transitions is ${T}_{1}\left(={\gamma }_{ij}^{-1}\right)=1ns$. The vibrational coherence time and electronic coherence dephasing time are ${T}_{2,vib}\left(={\Gamma }_{12}^{-1}={\Gamma }_{34}^{-1}\right)=1.7ns. {T}_{2,elec}\left(={\Gamma }_{13}^{-1}={\Gamma }_{14}^{-1}={\Gamma }_{23}^{-1}={\Gamma }_{24}^{-1}\right)=100 \,\mathrm{fs}$, respectively. The detuning parameter is defined as $\Delta ={\omega }_{e}-{\omega }_{31}$. The electric susceptibility of the molecular system corresponding to the applied field is introduced as^25^

4 $$\chi =\frac{2N\mathrm{\wp }}{{\varepsilon }_{o}{E}_{0}}\left({\rho }_{31}+{\rho }_{42}\right),$$

where $$N$$ is the molecular number density, which implies the concentration of the sample $$\left({C}_{p}\right)$$. Here electric susceptibility is a complex quantity $$\chi ={\chi }^{^{\prime}}+i{\chi }^{"}$$, and it’s real and imaginary part represents the refractive index and absorption coefficient of the system. In Fig. 2 we introduce three layers I, II, and III. Layers I and III are the walls of the cavity with the same nonmagnetic dielectric substance $$\left({\epsilon }_{1}=2.22\right)$$ and thickness $${d}_{1}=0.2\mu m$$, while the layer II has the dielectric constant $$\left({\epsilon }_{2}=1+\chi \right)$$ with thickness $${d}_{2}=5\mu m$$. It can be seen that the response of the molecular system to the applied field, appeared in electric susceptibility, imparts through the dielectric constant of layer II. The total thickness of the cavity is $$ L = 2d_{1} + d_{2}$$. Transfer matrix is defined as^26^

5 $$ M_{j} \left( {k_{2y} ,\omega_{e} ,d_{j} } \right) = \left( {\begin{array}{*{20}c} {\cos \left( {k_{2z}^{j} d_{j} } \right)} \\ {iq_{j} \sin \left( {k_{2z}^{j} d_{j} } \right)} \\ \end{array} \begin{array}{*{20}l} { i\sin \left( {k_{2z}^{j} d_{j} } \right)/q_{j} } \hfill \\ { \cos \left( {k_{2z}^{j} d_{j} } \right)} \hfill \\ \end{array} } \right),\quad j = 1. 2. 3 $$

where $${k}_{2z}^{j}=\left({\epsilon }_{j}{k}_{2}^{2}-{k}_{2y}^{2}\right)$$ is the $$z$$ component of the wavenumber, $${q}_{j}={k}_{2z}^{j}/{k}_{2}$$ , $${d}_{j}$$, indicate the thickness of the layer, and $$ j$$ shows the $$j$$ th layer of the medium. The wavenumber in a vacuum is $$ k_{2} = \omega_{e} /c$$. The wavenumber of the intracavity medium is given by $$ k_{2} = n_{2} \omega_{e} /c$$. For three layers’ model, the transfer matrix is given by

6 $$ Q\left( {k_{2y} ,\omega_{e} } \right) = M_{1} \left( {k_{2y} ,\omega_{e} ,d_{1} } \right)M_{2} \left( {k_{2y} ,\omega_{e} ,d_{2} } \right)M_{3} \left( {k_{2y} ,\omega_{e} ,d_{1} } \right). $$

By calculating matrix elements $Q_{ij} ,$ from the relation (6) and replacing them in the Fresnel equations, we reach the reflection ${\text{X}}_{r}$ and transmission ${\text{X}}_{t}$ as^27^

7 $$ \begin{aligned} {\text{X}}_{r} \left( {k_{2y} ,\omega_{e} } \right) & = \frac{{q_{o} \left[ {Q_{22} \left( {k_{2y} ,\omega_{e} } \right) - Q_{11} \left( {k_{2y} ,\omega_{e} } \right)} \right] - \left[ {q_{o}^{2} Q_{12} \left( {k_{2y} ,\omega_{e} } \right) - Q_{21} \left( {k_{2y} \omega_{e} } \right)} \right]}}{{q_{o} \left[ {Q_{22} \left( {k_{2y} ,\omega_{e} } \right) + Q_{11} \left( {k_{2y} ,\omega_{e} } \right)} \right] - \left[ {q_{o}^{2} Q_{12} \left( {k_{2y} ,\omega_{e} } \right) + Q_{21} \left( {k_{2y} ,\omega_{e} } \right)} \right]}}, \\ {\text{X}}_{t} \left( {k_{2y} ,\omega_{e} } \right) & = \frac{{2q_{o} }}{{q_{o} \left[ {Q_{22} \left( {k_{2y} ,\omega_{e} } \right) + Q_{11} \left( {k_{2y} ,\omega_{e} } \right)} \right] - \left[ {q_{o}^{2} Q_{12} \left( {k_{2y} ,\omega_{e} } \right) + Q_{21} \left( {k_{2y} ,\omega_{e} } \right)} \right]}}, \\ \end{aligned} $$

where $q_{o} = k_{2z} /k_{2}$ and $Q_{ij}$ denote the elements of matrix $Q\left( {k_{2y} ,\omega_{e} } \right)$ with the assumption ${\Delta }k_{2} \ll k_{2}$ and stationary phase theory, the reflection $\left( {{\text{X}}_{r} } \right)$ and transmission $\left( {{\text{X}}_{t} } \right)$ coefficients are introduced as ${\mathrm{X}}_{r}=\left|{\mathrm{X}}_{r}\right|exp\left(i{\varphi }_{r}\right)$ and ${\mathrm{X}}_{t}=\left|{\mathrm{X}}_{t}\right|exp\left(i{\varphi }_{t}\right)$. Finally, lateral or GH shifts for the reflected and transmitted laser field are obtained as

8 $${S}_{r,t}=-\frac{\lambda }{2\pi }\frac{d{\varphi }_{r,t}}{d\theta }.$$

After replacing the (7) into equations in relation (8), we obtain the lateral shifts in the reflected and transmitted laser field as^28^

9 $$ \begin{aligned} & S_{r} = - \frac{\lambda }{{2\pi \left| {{\text{X}}_{r} \left( {k_{2y} ,\omega_{e} } \right)} \right|^{2} }}\left\{ {Re\left[ {{\text{X}}_{r} \left( {k_{2y} ,\omega_{e} } \right)} \right]\frac{{dIm\left[ {{\text{X}}_{r} \left( {k_{2y} ,\omega_{e} } \right)} \right]}}{d\theta } - Im\left[ {{\text{X}}_{r} \left( {k_{2y} ,\omega_{e} } \right)} \right]\frac{{dRe\left[ {{\text{X}}_{r} \left( {k_{2y} ,\omega_{e} } \right)} \right]}}{d\theta }} \right\} \\ & {\text{and}} \\ & S_{t} = - \frac{\lambda }{{2\pi \left| {{\text{X}}_{t} \left( {k_{2y} ,\omega_{e} } \right)} \right|^{2} }}\left\{ {Re\left[ {{\text{X}}_{t} \left( {k_{2y} ,\omega_{e} } \right)} \right]\frac{{dIm\left[ {{\text{X}}_{t} \left( {k_{2y} ,\omega_{e} } \right)} \right]}}{d\theta } - Im\left[ {{\text{X}}_{t} \left( {k_{2y} ,\omega_{e} } \right)} \right]\frac{{dRe\left[ {{\text{X}}_{t} \left( {k_{2y} ,\omega_{e} } \right)} \right]}}{d\theta }} \right\}. \\ \end{aligned} $$

In the following, we analyze the sensitivity of the designed model; the sensitivity of the sensor is defined by GH shift dependence on the refractive index change as

10 $$S= \frac{\Delta {S}_{r max}}{\Delta {n}_{2}}.$$

Here, $\Delta {S}_{r}$ defines as the maximum amount of the changed GH shift for the reflected laser field, and $\Delta {n}_{2}$ refers to the variation of the refractive index of intracavity area.

## Results and discussion

**Figure 3:**
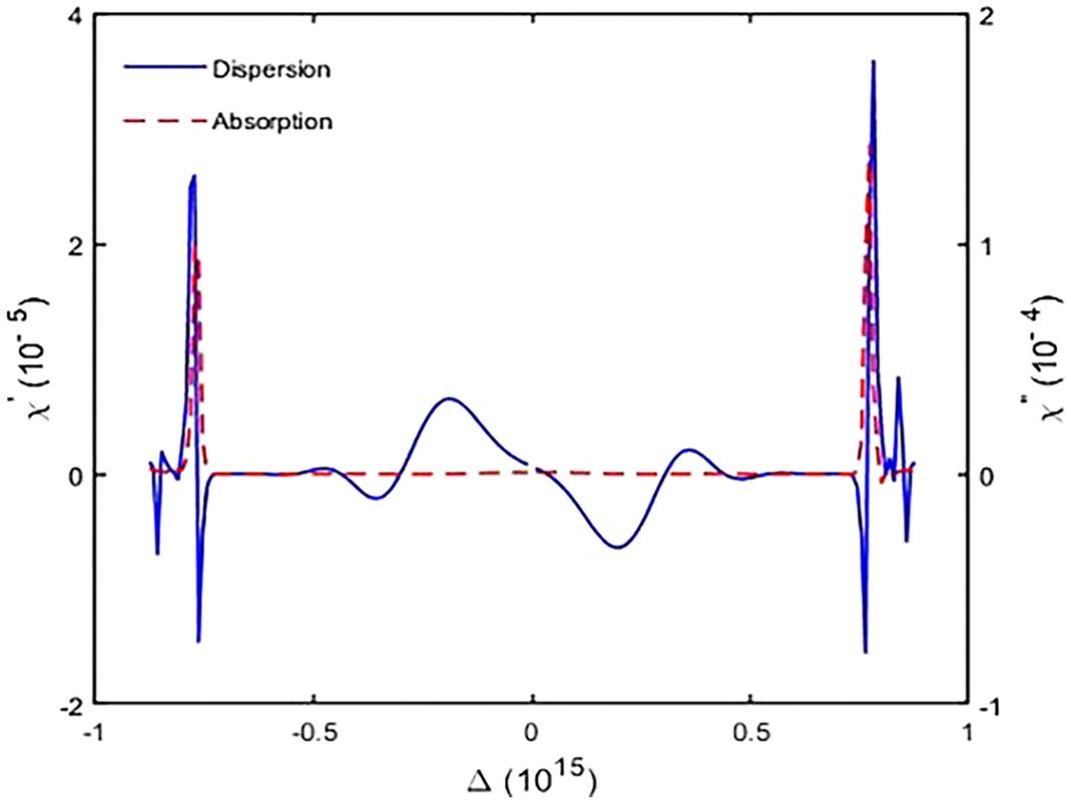
Real (solid) and imaginary (dashed) parts of the susceptibility versus laser field detuning for ${\Gamma }_{12}^{-1}={\Gamma }_{34}^{-1}=1.7 \mathrm{ ps}, {\Gamma }_{13}^{-1}={\Gamma }_{14}^{-1}={\Gamma }_{23}^{-1}={\Gamma }_{24}^{-1}=100 \,\mathrm{fs}, {\gamma }_{ij}^{-1}=1 \,\mathrm{ns}, \tau =17 \,\mathrm{fs}$, and ${\varphi }^{"}={-10}^{3} {\,\mathrm{fs}}^{2}$.

**Figure 4:**
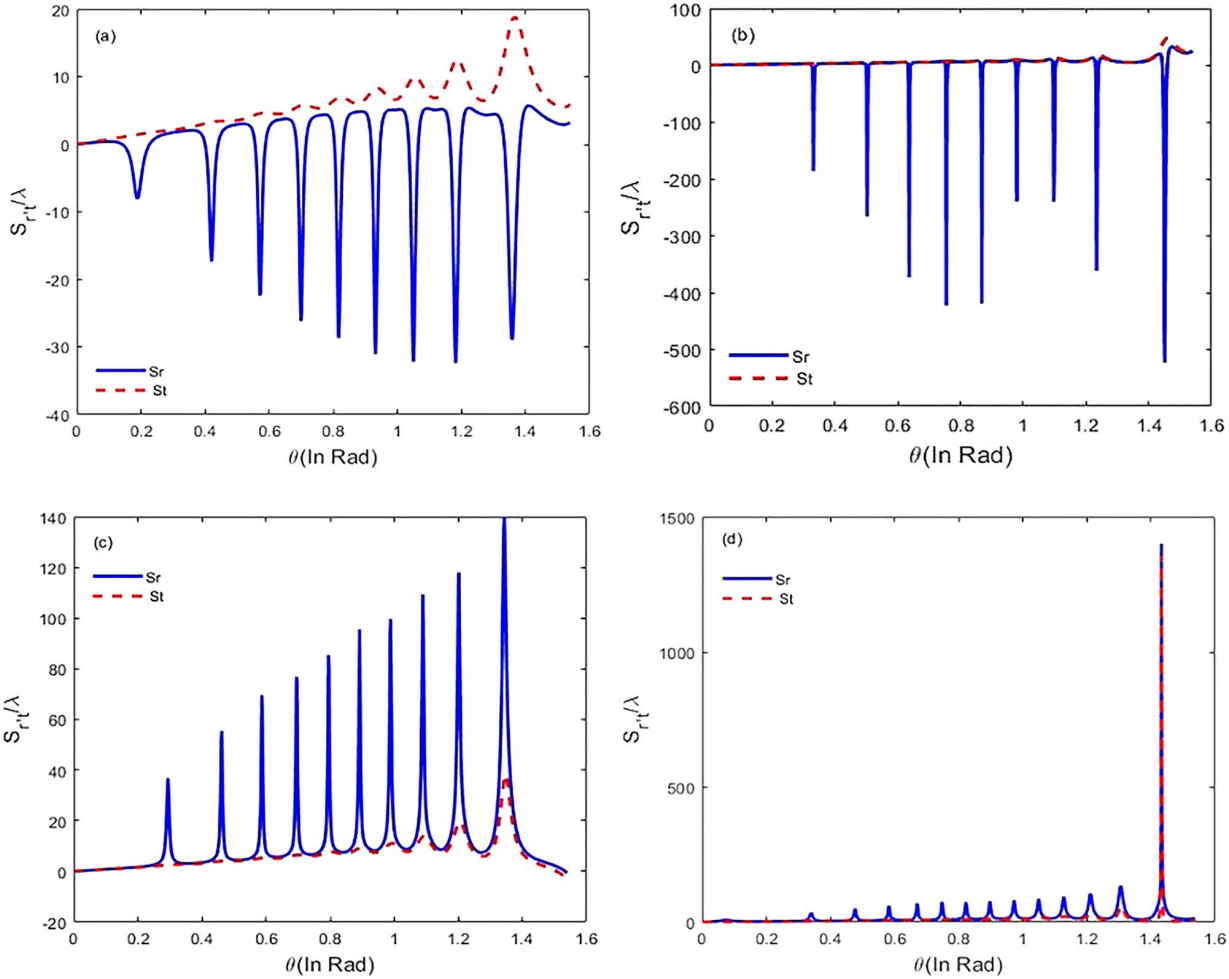
Lateral shifts $${S}_{r}$$ (solid) and $${S}_{t} $$(dashed) at different incident angles ranging from 0 to $$\pi /2$$ rad for $${\Gamma }_{12}^{-1}={\Gamma }_{34}^{-1}=1.7 \mathrm{ps}, {\Gamma }_{13}^{-1}={\Gamma }_{14}^{-1}={\Gamma }_{23}^{-1}={\Gamma }_{24}^{-1}=100 \,\mathrm{fs}, {\gamma }_{ij}^{-1}=1 \,\mathrm{ns}, \tau =17 \,\mathrm{fs}$$, $${\varphi }^{"}={+10}^{3}{ \,\mathrm{fs}}^{2}$$ (**a**) $$\Delta =2500 \,{\mathrm{cm}}^{-1},$$ (**b**) $$\Delta =2600 \,{\mathrm{cm}}^{-1},$$ (**c**) $$\Delta =2700 \,{ \mathrm{cm}}^{-1},$$ and (**d**) $$\Delta =2800 \,{\mathrm{cm}}^{-1}.$$

**Figure 5:**
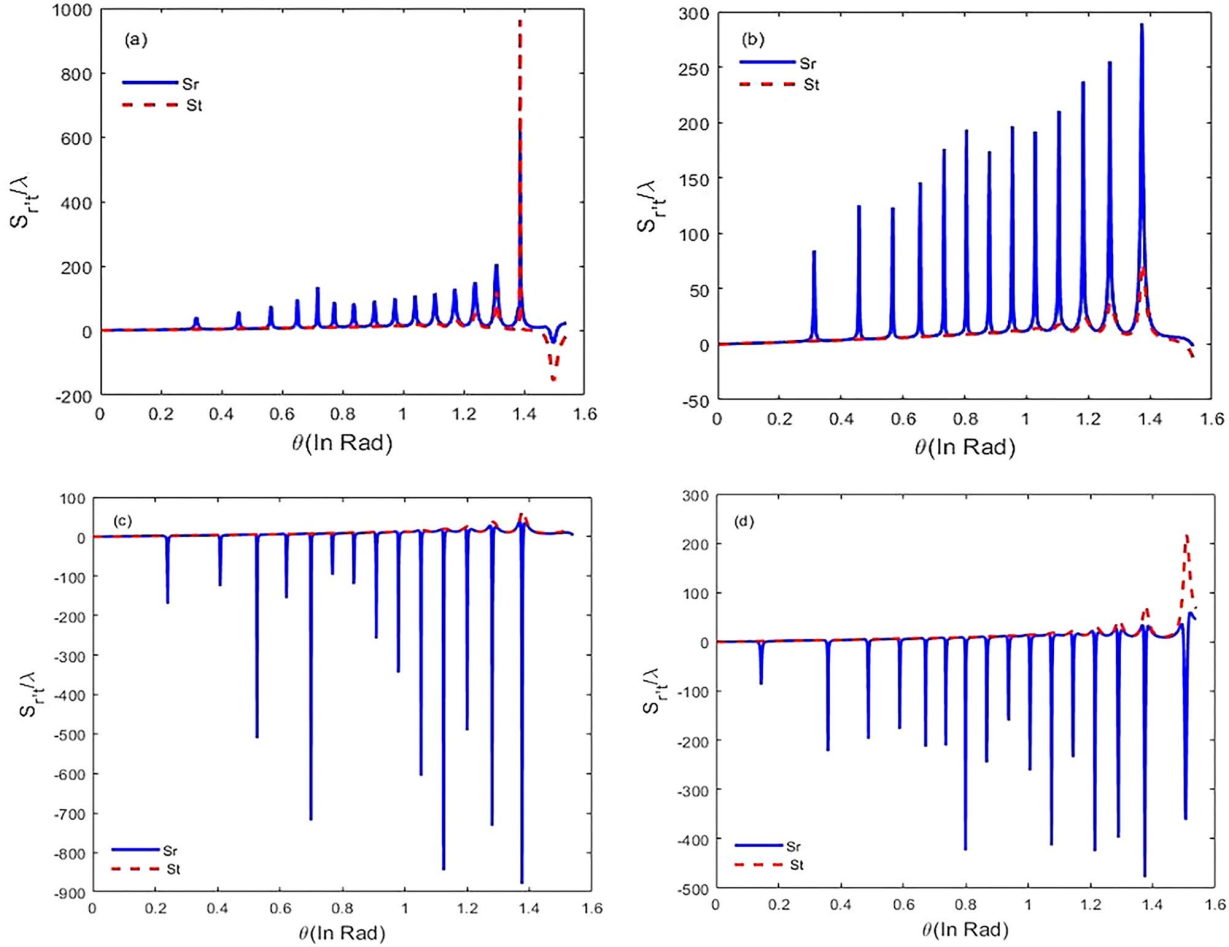
Lateral shifts ${S}_{r}$ (solid) and ${S}_{t}$(dashed) at different incident angles ranging from 0 to $\pi /2$ rad for ${\Gamma }_{12}^{-1}={\Gamma }_{34}^{-1}=1.7 \mathrm{ps}, {\Gamma }_{13}^{-1}={\Gamma }_{14}^{-1}={\Gamma }_{23}^{-1}={\Gamma }_{24}^{-1}=100 \,\mathrm{fs}, {\gamma }_{ij}^{-1}=1 \,\mathrm{ns}, \tau =17 \,\mathrm{fs}$, and ${\varphi }^{"}={-10}^{3} {\,\mathrm{fs}}^{2}$ (**a**) $\Delta =2900 \,{\mathrm{cm}}^{-1},$ (**b**) $\Delta =3000 \,{\mathrm{cm}}^{-1},$ (**c**) $\Delta =3100 \,{\mathrm{cm}}^{-1},$ and (**d**) $\Delta =3200 \,{\mathrm{cm}}^{-1}.$

We first solve Eq. (3) numerically in steady-state to obtain the susceptibility of the system via relation (4). Then, we discuss the GH shifts of the transmitted and reflected single-chirped laser pulse via Eq. (9). In Fig. 3 we show the dispersion and absorption properties of chirped laser pulse versus chirped laser field detuning. The selected parameters are ${\varphi }^{"}={-10}^{3}{fs}^{2}$, ${\Gamma }_{12}^{-1}={\Gamma }_{34}^{-1}=1.7 ps$, ${\Gamma }_{13}^{-1}={\Gamma }_{14}^{-1}={\Gamma }_{23}^{-1}={\Gamma }_{24}^{-1}=100 \,\mathrm{fs}$, ${\gamma }_{ij}^{-1}=1 \mathrm{ns }\tau =17 \,\mathrm{fs}$,$N=6.5\times {10}^{12 } \,{\mathrm{molecule}}/{\mathrm{cm}}^{3}$. According to Fig. 3, we observe that for negative chirped laser field with chirp spectrum ${\varphi }^{"}=-{10}^{3}{fs}^{2},$ two peaks in the absorption spectrum appear that correspond to vibrational level splitting in the excited state. Next, we change the detuning of the laser field from $2500 \,{\mathrm{cm}}^{-1}$ to 2800 ${cm}^{-1}$ by steps $100\,{\mathrm{ cm}}^{-1}$ and plot GH shifts of the reflected and the transmitted chirped laser pulse against incident angle $\theta$ ranging from $\theta =0$ to $\theta =\pi /2 \mathrm{ rad}$ (Fig. 4). For $\Delta =2500 \,{\mathrm{ cm}}^{-1}, {\varphi }^{"}=+{10}^{3}{fs}^{2},$ there are negative GH shifts for the reflected laser field while the GH shifts for transmitted beam are positive (Fig. 4a).When the detuning of laser field changes to $\Delta =2600 \,{\mathrm{cm}}^{-1},$ the behavior is similar, but there are significant negative GH shifts for the reflected laser field at the specific incident angles. In this case, GH shifts for the transmitted laser field are almost zero (Fig. 4b). For $\Delta =2700 \,{\mathrm{cm}}^{-1},$ the reflected laser field endures positive shifts, whereas the transmitted laser field remains positive (Fig. 4c). Hence, we conclude that with increasing $\Delta =2600 \,{\mathrm{cm}}^{-1}$ to $\Delta =2700 \,{\mathrm{cm}}^{-1}$, lateral shifts for reflected laser field can be switched from negative to positive. By increasing detuning of the chirped laser field, GH shifts for both the reflected and transmitted laser field remain positive. Finally, for $\Delta =2800 \,{\mathrm{cm}}^{-1},$ at $\theta =1.43 \mathrm{rad},$amount of lateral shifts for both the reflected and transmitted laser field increases to ${S}_{r}=1.39\times {10}^{3}\lambda$, ${S}_{t}=1.35\times {10}^{3}\lambda$(Fig. 4d). Next, it is interesting to investigate negative chirped laser pulse behavior with chirp spectrum ${\varphi }^{"}=-{10}^{3}{fs}^{2},$ so we change the detuning of laser field from $\Delta =2900\,{ \mathrm{cm}}^{-1}$ to $\Delta =3200 \,{\mathrm{cm}}^{-1}$ by steps $100 \,{\mathrm{cm}}^{-1}$ and plot GH shifts for both the reflected and transmitted laser field based on incident angles from $\theta =0$ to $\theta =\pi /2 \mathrm{rad}$. The results show that by increasing the detuning of the laser field, GH shifts for both beams have increased significantly. In Fig. 5a, for $\Delta =2900 \,{\mathrm{cm}}^{-1},$ there are positive GH shifts for both the reflected and transmitted beams until $\theta =1.38 \mathrm{ rad}$, but for angles greater than $\theta =1.38 rad$, the displacements become negative. The magnitude of lateral shifts for the reflected and transmitted laser field at $\theta =1.38 \mathrm{ rad},$ is ${S}_{r}=623.90\lambda , {S}_{t}=963.26 \lambda$. According to Fig. 5b, for $\Delta =3000\,{ \mathrm{cm}}^{-1},$ lateral shifts for both reflected and transmitted laser fields are positive, and displacements for several incident angles have increased. For $\Delta =3100 \,{\mathrm{cm}}^{-1},$ there are huge lateral shifts for reflected laser field at some incident angles. A large shift is observed at $\theta =1.38 \mathrm{rad},$ and the amount of this lateral shift is ${S}_{r}=-878.13\lambda$. For transmitted laser field, GH shifts are nearly zero (Fig. 5c). For $\Delta =3200\,{ \mathrm{cm}}^{-1},$ lateral shifts for reflected laser field (transmitted laser field) are negative (positive), and the largest lateral shift is observed at $\theta =1.38 \mathrm{ rad}$ (Fig. 5d). In this study, we focus on maximum GH shifts for the reflected laser field that are sensitive to refractive index variation of the intracavity area. Based on the obtained result, for positive chirped laser pulse, maximum GH shifts for the reflected laser field are observed in $\Delta =2600\,{ \mathrm{cm}}^{-1}$, while GH shifts for transmitted laser field are almost negligible (Fig. 4b). For negative chirped laser pulse, maximum GH shifts for the reflected laser field is observed in $\Delta =3100 \,{\mathrm{cm}}^{-1}$ (Fig. 5c).

**Figure 6:**
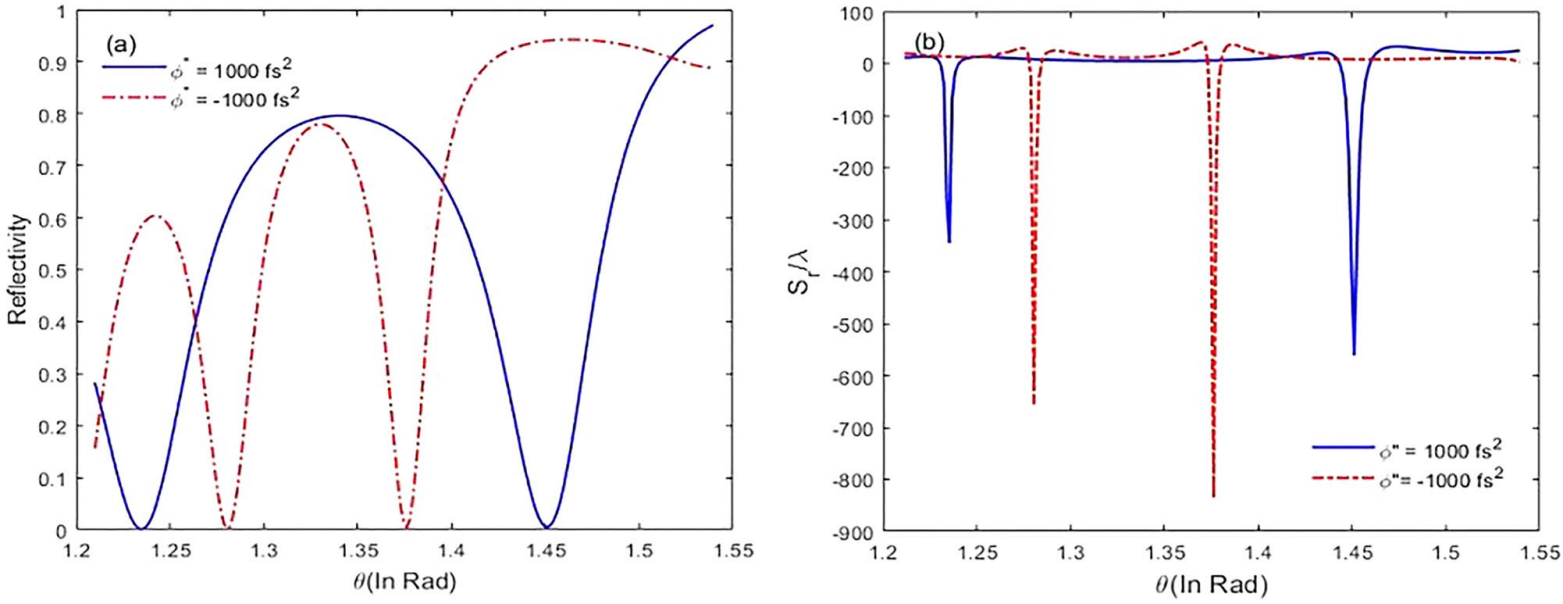
The reflectivity (**a**) and reflected lateral shifts (**b**) for negative and positive chirped laser field versus different incident angles for ${\Gamma }_{12}^{-1}={\Gamma }_{34}^{-1}=1.7 \mathrm{ps}, {\Gamma }_{13}^{-1}={\Gamma }_{14}^{-1}={\Gamma }_{23}^{-1}={\Gamma }_{24}^{-1}=100 \,\mathrm{fs}, {\gamma }_{ij}^{-1}=1 \,\mathrm{ns}, \tau =17 \,\mathrm{fs}$, and ${\varphi }^{"}={\pm 10}^{3} {\,\mathrm{fs}}^{2}.$

**Figure 7:**
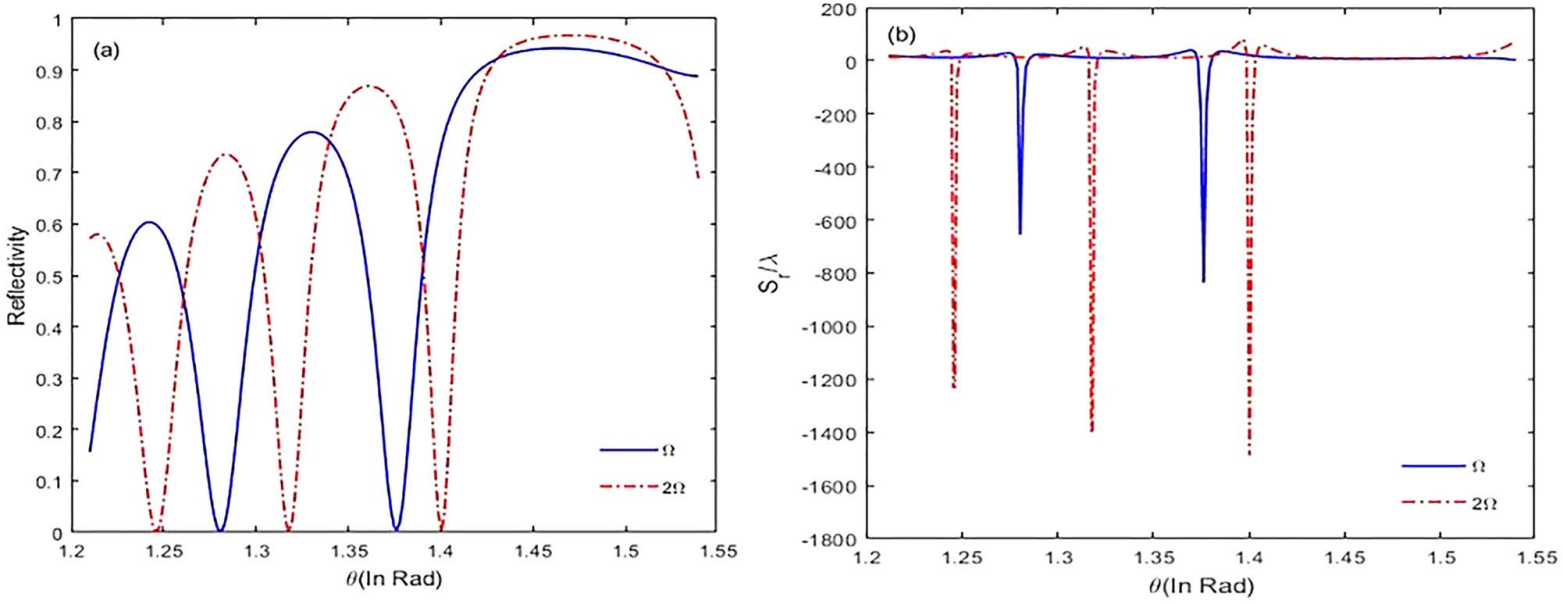
The reflectivity (**a**) and reflected lateral shifts (**b**) for negative chirped laser field versus different incident angles by increasing the intensity of laser field from $\Omega$ to $2\Omega$ for ${\Gamma }_{12}^{-1}={\Gamma }_{34}^{-1}=1.7 \mathrm{ps}, {\Gamma }_{13}^{-1}={\Gamma }_{14}^{-1}={\Gamma }_{23}^{-1}={\Gamma }_{24}^{-1}=100 \,\mathrm{fs}, {\gamma }_{ij}^{-1}=1 \,\mathrm{ns}, \tau =17 \,\mathrm{fs}$, and ${\varphi }^{"}={-10}^{3}{fs}^{2}$**.**

In Fig. 6, we plot reflectivity ($R={\left|{\chi }_{r}\right|}^{2}$) and GH shifts versus incident angles for positive and negative chirped laser field. With decreasing reflectivity, guiding mode in the cavity is excited, and the incident light is coupled into the cavity. According to Fig. 6a, for ${\varphi }^{"}=-{10}^{3}{fs}^{2},$ by increasing the incident angle, the peak of reflectivity is narrowing. Thus, the corresponding GH shift increases, and it appears at a smaller angle (Fig. 6b). This is due to the strong coupling of the laser field into the cavity. Also, it is related to the deeper penetration of light along z-axis. So, giant GH shits in special incident angles is proportional to strong deep penetration. In Fig. 7, we study the intensity of the applied field on the GH shifts. In this regard, we focus on negative chirped laser pulse with ${\varphi }^{"}=-{10}^{3}{fs}^{2}$, and alter the intensity of the applied field. Figure 7a displays the reflectivity of laser pulse versus incident angles. It is evident that with increasing the intensity of the applied field, the peak of reflectivity is narrowing, and it appears at a big incident angle. Appropriately, the GH shifts and coupling of the laser field into the cavity are increased (Fig. 7b). In addition, maximal lateral shifts are related to the negative chirped laser pulse.

**Figure 8:**
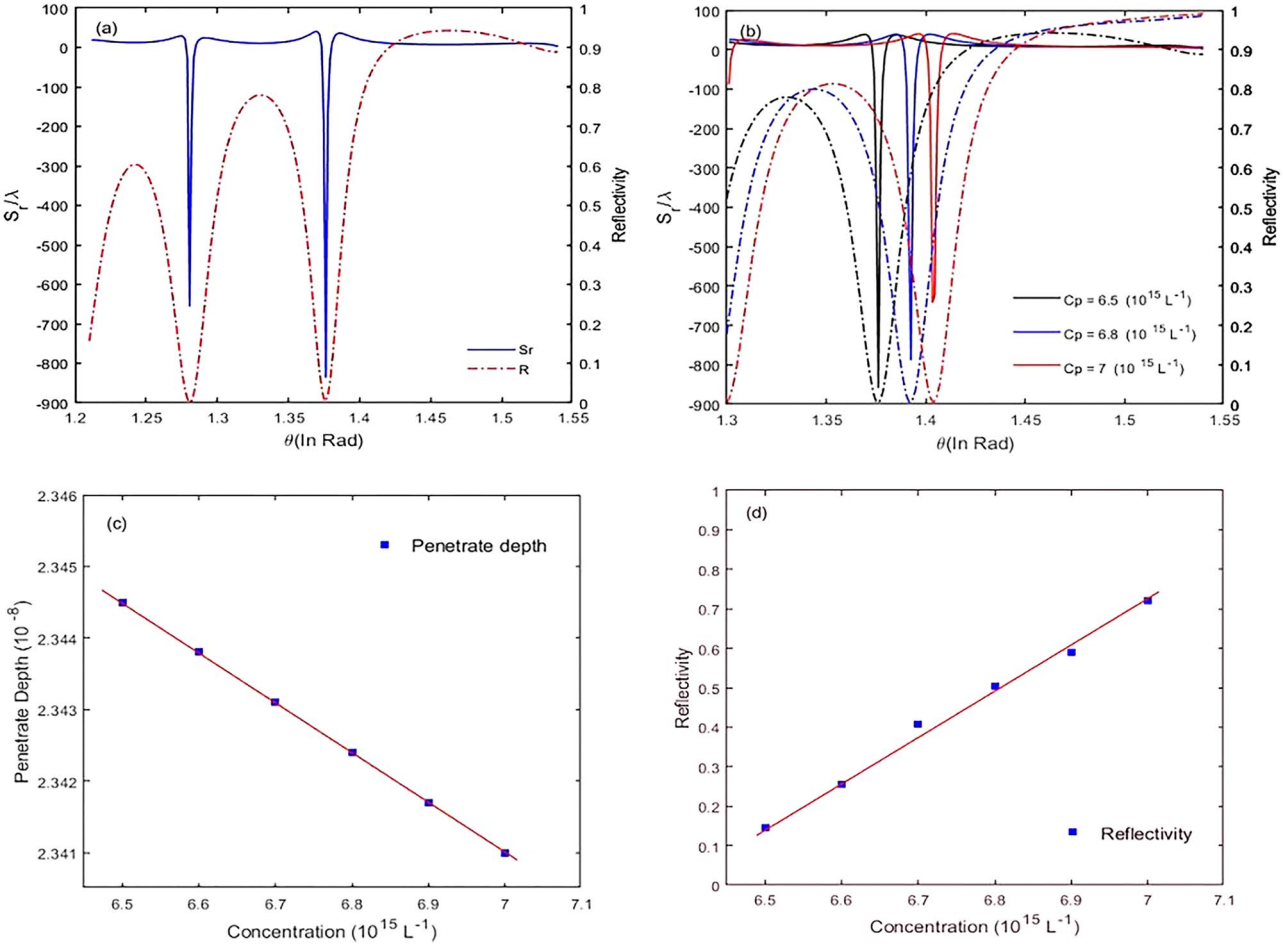
Reflected lateral shifts, ${S}_{r}$, and reflectivity versus incident angles (**a**), Lateral shifts ${S}_{r}$, and reflectivity versus incident angles for different concentration (**b**), Penetrate depth (**c**), Reflectivity (**d**) for negative chirped laser field versus concentration for ${\Gamma }_{12}^{-1}={\Gamma }_{34}^{-1}=1.7\mathrm{ ps}, {\Gamma }_{13}^{-1}={\Gamma }_{14}^{-1}={\Gamma }_{23}^{-1}={\Gamma }_{24}^{-1}=100\mathrm{ fs}, {\gamma }_{ij}^{-1}=1\mathrm{ ns}, \tau =17\mathrm{ fs}$, and ${\varphi }^{"}={-10}^{3 }{\,\mathrm{fs}}^{2}$, and $\Delta =3100 \,{\mathrm{cm}}^{-1}.$

In Fig. 8a, we plot GH shifts of the reflected laser field and reflectivity against incident laser field angles. When the laser field is incident upon the sensor, at special incident angles, the reflectivity reaches a minimum, and the maximum GH shift appears. In Fig. 8b, we change the concentration of sample $\left({C}_{p}\right)$, i.e., N, and plot GH shift for reflected laser field and reflectivity versus incident angles. The result shows that by increasing the concentration, the GH shift for reflected laser field reduces and shifts to large angles. This means that, the laser field is weakly coupled into the cavity, and a constant reflectivity is shifted to large angles. Note, reflectivity at $\theta =1.38\, \mathrm{rad}$ is increased. In Fig. 8c, we plot penetrate depth versus concentration of the sample in a constant incident angle $\theta =1.38 \mathrm{rad }\left(d=\frac{\lambda }{4\pi }{\left({{n}_{1}}^{2}{sin\theta }^{2}-{{n}_{2}}^{2}\right)}^{-1/2}\right)$^29^. When the concentration of sample increases, penetrate depth accordingly reduces, thus the incident laser field cannot be strongly coupled into the cavity. So, the GH shift significantly decreases. Furthermore, the result shows that by increasing concentration of the sample, in a constant incident angle $\theta =1.38 \,{\rm rad}$ reflectivity for negative chirped laser field increases (Fig. 8d). Note that short penetration depth is the key issue and forms the basis for many sensing devices.

Note that the susceptibility of the molecular system corresponding to the applied field is given by Eq. (4), where $N$ is the molecular number density, which implies the concentration of the sample $\left({C}_{p}\right)$. In all simulations, number concentration is chosen as $N=6.5\times {10}^{12 } \,{\mathrm{molecule}}/{\mathrm{cm}}^{3}$. This quantity is relatively large, and is proportional to the thickness of the cavity (${d}_{2}=5 \,\upmu{\mathrm{m}})$. According to Fig. 8b, by increasing number concentration from $6.5\times {10}^{12 } \,{\mathrm{molecule}}/{\mathrm{cm}}^{3}$ to $7\times {10}^{12 } \,{\mathrm{molecule}}/{\mathrm{cm}}^{3}$, reflectivity increases, but the GH shift for reflected laser field reduces. Also, according to Fig. 8c, penetrate depth reduces. So, the results show that this sensor can be worked in a low number concentration, i.e., $N=6.5\times {10}^{12 } \,{\mathrm{molecule}}/{\mathrm{cm}}^{3},$ better than in large number density.

**Figure 9:**
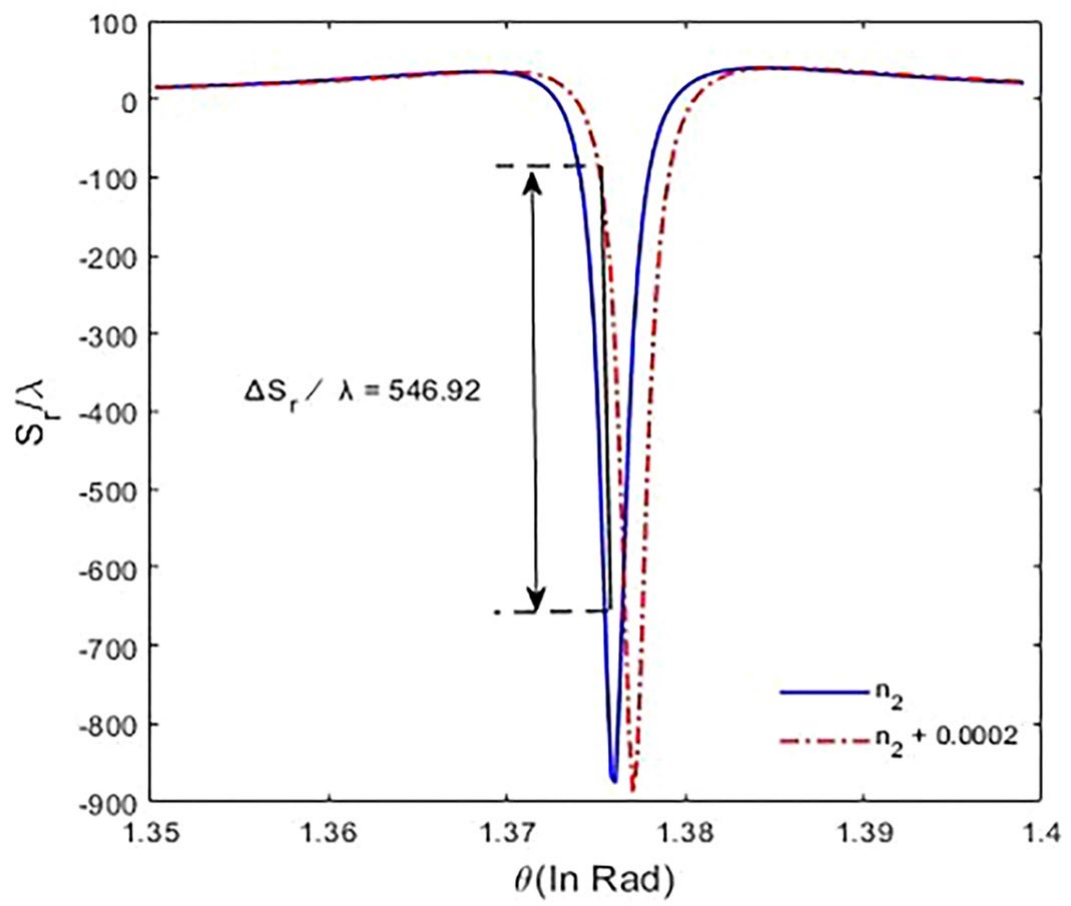
Variation of GH shift versus incident angle by changing the refractive index of intracavity medium for ${\Gamma }_{12}^{-1}={\Gamma }_{34}^{-1}=1.7\mathrm{ ps}, {\Gamma }_{13}^{-1}={\Gamma }_{14}^{-1}={\Gamma }_{23}^{-1}={\Gamma }_{24}^{-1}=100\mathrm{ fs}, {\gamma }_{ij}^{-1}=1\mathrm{ ns}, \tau =17\mathrm{ fs}$,${\varphi }^{"}={-10}^{3} {\,\mathrm{fs}}^{2}$, and $\Delta {n}_{2}=2\times {10}^{-4}.$

In fine, we discuss the sensitivity of GH shifts for the proposed biosensor. So, we alter the refractive index of the intracavity containing sample and plot GH shifts for the reflected laser field based on different incident angles. According to relation (10), for ${\varphi }^{"}=-{10}^{3}{fs}^{2}, \Delta =3100 \,{\mathrm{cm}}^{-1}$, and $\Delta {n}_{2}$=$2\times {10}^{-4}$, the maximum value of the changed GH shift for the reflected laser field, and the sensitivity are $546.92\lambda$ and $2.7\times {10}^{6}\lambda$, respectively (Fig. 9).

## Conclusions

We propose a four-level molecular system with two vibrational states that interact with a single chirped laser field. The susceptibility of the system is calculated by the density matrix method, while the GH shifts of the reflected and transmitted laser field is obtained by the transfer matrix method. We discuss the GH shifts for the reflected and transmitted chirped laser field against incident angle for several laser field detuning. The influence of the positive and negative chirped laser field on GH shifts are also discussed. It is proven that the coupling of the laser field for the negative chirped laser field is strong, and the giant GH shifts for the reflected laser field are observed for the negative chirped laser field. Furthermore, when we increase the intensity of the laser field, the GH shifts for reflected laser field at specific incident angles are increased significantly. Also, we conclude that by increasing the concentration of the sample, GH shifts and penetrate depth reduces. So, in this sensor, a strong focus has been made on the detection of substances in low concentrations of the cavity medium. The results show that in the proposed biosensor, the maximum sensitivity reaches $2.7\times {10}^{6}\lambda$. Note that the GH shift basically depends on the polarization and refractive index of the medium. In the present work, we show that it also depends on the concentration of the sample and the vibrational frequencies. The polarization of the medium is definitely depending on the intensity of the laser field.

Finally, we emphases that there is some relation between the present proposal with the previous studies, but what makes our proposal more important and also different than the previous works are as follows:

1. In previous works, this model has already been studied for other purposes. References^25,26^ imply the calculation methods for the medium susceptibility and transfer matrix. In ref.^27^, authors studied the lateral shift of a light beam reflecting from a dielectric slab by a metal, or in ref.^28^ authors investigated negative lateral shift of a light beam transmitted through a dielectric slab. It is found that when a light beam travels through a slab of the optically denser dielectric medium, the lateral shift of the transmitted beam can be negative.
2. In our proposal, a four-level molecular system is used just by one chirped laser pulse to couple ground vibrational states to excited vibrational states. This is a simple method compared to other methods, where two or more laser fields (pump and probe) has been used for light mater interaction. Furthermore, according to our acknowledge, this model has not yet been investigated with a chirped laser pulse for a four-level molecular system such as an oxazine. In addition, the set of the density matrix equations for a four-level molecular system with a chirped laser pulse are solved by Laplace transformation in the study-state. While, in previous works for the molecular systems, the dynamical response of the population transfer and vibrational coherence have usually been proposed. Furthermore, the sensor behavior of this molecular medium by a negative/positive chirped laser field has not been proposed.
3. We record the refractive index of the sample directly by calculating optical susceptibility. It has been observed that the optical susceptibility of the medium can be changed by using different parameters of the chirped laser field. This variation modifies the resonant condition of the cavity, and therefore the manipulation of the field on the lateral shifts could be observed. This can be considered as a new method for sensing compared to previous methods. In fact, this paper introduces an optical sensor with a high sensitivity of $2.7\times {10}^{6}\lambda$, compared to the other recent studies^9,18,30^.

## References

[1] Fine GF, Cavanagh LM, Afonja A, Binions R (2010). Metal oxide semi-conductor gas sensors in environmental monitoring. Sensors.

[2] Reiner JE (2012). Disease detection and management via single nanopore-based sensors. Chem. Rev..

[3] Patel PD (2002). (Bio)sensors for measurement of analytes implicated in food safety: a review. TrAC Trends Anal. Chem..

[4] Fang Y (2006). Label-free cell-based assays with optical biosensors in drug discovery. Assay Drug Dev. Technol..

[5] Lahijani, B. V. *et al.* Optical surface waves on one-dimensional photonic crystals: investigation of loss mechanisms and demonstration of centimeter-scale propagation. *Phys. Opt.* arXiv:1907.00187 (2019).

[6] Huang YH, Ho HP, Kong SK, Kabashin AV (2012). Phase-sensitive surface plasmon resonance biosensors: Methodology, instrumentation and
applications. Ann. Phys..

[7] Sreekanth KV (2018). Biosensing with the singular phase of an ultrathin metal-dielectric nanophotonic cavity. Nat. Commun..

[8] Sreekanth KV (2018). Supporting information large-area silver-stibnite nanoporous plasmonic films for label-free biosensing. ACS Appl. Mater. Interfaces.

[9] Sreekanth KV (2019). Phase-change-material-based low-loss visible-frequency hyperbolic metamaterials for ultrasensitive label-free biosensing. Adv. Opt. Mater..

[10] Yan, R., Wang, T., Jiang, X., Zhong, Q. & Huang, X. Extreme sensitivity refractive index sensor based on lithography-free metal-dielectric cavity. *Phys. Opt.* arXiv:1907.11858 (2019).

[11] Goos F, Lindberg-Hänchen H (1949). Neumessung des Strahlversetzungseffektes bei Totalreflexion. Ann. Phys..

[12] Goos F, Lindberg-Hänchen H (1947). Ein neuer und fundamentaler versuch zur total reflexion. Ann. Phys..

[13] Munshid M (2017). Design and implementation of heterodyne detection based on photonic crystal fiber sensor. Appl. Sci..

[14] Sakata T, Togo H, Shimokawa F (2000). Reflection-type 2×2 optical waveguide switch using the Goos–Hänchen shift effect. Appl. Phys. Lett..

[15] Sreekanth KV, Zeng S, Yong KT, Yu T (2013). Sensitivity enhanced biosensor using graphene-based one-dimensional photonic crystal. Sens. Actuators B Chem..

[16] Nie Y (2014). Detection of chemical vapor with high sensitivity by using the symmetrical metal-cladding waveguide-enhanced Goos-Hänchen shift. Opt. Express.

[17] Chen C (2007). Goos: optical temperature sensing based on the Goos–Hänchen effect. Appl. Opt..

[18] Yin L (2020). High sensitivity terahertz biosensor based on Goos–Hänchen effect in graphene based on goos-hänchen effect in graphene. IEEE Photonics J..

[19] Zhang Z, Yang X, Yan X (2013). Population transfer and generation of arbitrary superpositions of quantum states in a four-level system using a single-chirped laser pulse. J. Opt. Soc. Am. B.

[20] Wan Y, Cheng M, Zheng Z, Liu K (2019). Polarization-modulated, Goos–hanchen shift sensing for common mode drift suppression. Sensors (Switzerland).

[21] Kop RHJ, Sprik R (1995). Phase-sensitive interferometry with ultrashort optical pulses. Rev. Sci. Instrum..

[22] Sun J (2012). Optical transduction of E. Coli O157:H7 concentration by using the enhanced Goos–Hänchen shift. J. Appl. Phys..

[23] Buckup T, Hauer J, Serrat C, Motzkus M (2008). Control of excited-state population and vibrational coherence with shaped-resonant and near-resonant excitation. J. Phys. B At. Mol. Opt. Phys..

[24] Afa IJ, Serrat C (2016). Quantum control of population transfer and vibrational states via chirped pulses in four level density matrix equations. Appl. Sci..

[25] Scully MO, Zubairy S (1997). Quantum Optics.

[26] Liu N, Zhu SY, Chen H, Wu X (2002). Superluminal pulse propagation through one-dimensional photonic crystals with a dispersive defect. Phys. Rev. E - Stat. Phys. Plasm. Fluids Relat. Interdiscip. Top..

[27] Wang L-G, Chen H, Liu N-H, Zhu S-Y (2006). Negative and positive lateral shift of a light beam reflected from a grounded slab. Opt. Lett..

[28] Li CF (2003). Negative lateral shift of a light beam transmitted through a dielectric slab and interaction of boundary effects. Phys. Rev. Lett..

[29] Li Q, Zhang B, Shen J (2013). Goos–Hänchen shifts of reflected terahertz wave on a COC–air interface. Opt. Express.

[30] Zhang L, Farhat M, Nabil Salama K (2020). Spectrometer-free graphene plasmonics based refractive index sensor. Opt. Express.

